# 

**Published:** 1917-02-03

**Authors:** 


A number of Leeds charities benefit under the will of Miss Caroline Lambert, of Headingley, Leeds. Amongst the legacies are the following: General Infirmary, £1,000; Hospital for Women and Children, £500; District Nursing Association, £250; and the Tuberculosis Association, £250.

### Royal British College of Nursing

### MEMBERS OF THE COUNCIL.

SINCE we published the names of the Council of the College of Nursing in our issue of October 28, 1916, page 75, the following have been added as members:—

Miss Graham, Matron Trained Nurses' Institute, Edinburgh.
Dr. D. J. Mackintosh, M.B., M.V.O., Superintendent Western Infirmary, Glasgow.
Professor James Ritchie, M.D., Chairman Scottish Board.

The following members have now been added on the amalgamation of the Royal British Nurses' Association with the College of Nursing:—

Miss M. E. Belsham (Matron London Homœopathic Hospital).
Miss Emma Alice Cattell (Private Nurse).
Miss Cornelia Cave Browne-Cave (District Nurse, North London District Nursing Association).
Miss Mary C. Good (Private Nurse).
Mrs. Josephine Latter (late Matron Chelsea Infirmary).
Miss Eveline Marcon (Sister-in-Charge, Trained Nurses' Institute, St. Bartholomew's Hospital).
Mr. Herbert J. Paterson, M.C., M.A., F.R.C.S.
Miss M. C. Sinzinine (Matron Queen Alexandra's Hospital for Officers).
Miss Constance E. Thomson (late Sister, Middlesex Hospital).
Dr. W. Bezly Thorne, M.D., M.R.C.P.
Miss Elizabeth L. Probert Williams (Private Nurse).
Miss E. A. Montgomery Wilson, R.R.C. (Matron, King Edward VII. Hospital, Cardiff).

### Additions to Conditions of Registration.

To the Conditions of Registration applicable during the period of grace to existing nurses the following two new conditions have been added to those of the College of Nursing, Limited, published in our issue of October 28, p. 75:—

"*Nurses Trained after 1900*," add after 2 (*d*):—(*e*) In deciding upon applications of candidates whose training does not fulfil the above conditions, the Council may have regard to exceptional experience obtained subsequent to qualification, or to professional status.

"*Institutions Recognised for Training*," add after 5 (*c*):—(*d*) Or such Schools as are recognised at the present date (December 1916) by the Local Government Board as Training Schools.

Rates of Subscription: Twelve months, 6/6; Six months, 4/-; Three months, 2/-; post free to any address in the United Kingdom. Abroad, Twelve months, 8/8; Six months, 5/-; Three months, 2/6, post free. The Hospital "Index" to Volume LX., April to September 1916, is now ready, price 2½d. per copy, post free. Address The Manager, "The Hospital," The Hospital Building, 28/29 Southampton Street, Strand, London, W.C.

